# Supplementary material for: WordCommentsAnalyzer: A windows software tool for qualitative research
Source: F1000Res. 2018 Sep 4;7:536. Originally published 2018 May 3. [Version 2] doi: 10.12688/f1000research.14819.2 (PMC6137411; doi:10.12688/f1000research.14819.2)
Supplement: Supplementary file 3 [file f1000research-7-17614-s0002.tgz › b2a79496-4ada-47c4-9682-a99ce867836d.zip!/Eight Iranian Health Journals/Journal of Health System Research _4.docx]

© Journal of Health System Research . This work is licensed under CC BY-NC 4.0 (https://creativecommons.org/licenses/by-nc/4.0/).

Volume & Issue: دوره 13، شماره 2، 1396

Title: بررسی تأثیر کلاس‌های آمادگی زایمان فیزیولوژیک در دوران بارداری بر خودکارامدی زنان در زایمان طبیعی در مراجعان مراکز منتخب شهر اصفهان

http://hsr.mui.ac.ir/index.php/jhsr/article/view/2848

مقدمه: خودکارامدی، اعتقاد شخص به توانایی‌های خود برای انجام یک رفتار خاص به صورت موفقیت‌آمیز می‌باشد که می‌تواند بر رفتار، الگوهای فکری و عکس‌العمل‌های او تأثیر بگذارد. مادرانی که خودکارامدی بالایی دارند، با غلبه بر ترس زایمان، انجام آن را دست یافتنی می‌دانند. بنابراین، مطالعه حاضر با هدف تعیین تأثیر کلاس‌های آمادگی زایمان فیزیولوژیک بر خودکارامدی زنان جهت زایمان طبیعی انجام شد. روش‌ها: این مطالعه از نوع کارآزمایی بالینی دو گروهی و یک سوکور بود که بر روی 72 مادر باردار مراجعه کننده به مراکز بهداشتی- درمانی منتخب شهر اصفهان در سال 1393 انجام گردید. نمونه‌ها به روش تخصیص تصادفی در دو گروه قرار گرفتند. افراد گروه مداخله کلاس‌های آموزش زایمان فیزیولوژیک را به صورت 8 جلسه 2 ساعته دریافت نمودند. افراد گروه شاهد نیز از مراکزی انتخاب شدند که کلاس‌های آموزش زایمان فیزیولوژیک نداشتند. ابزار جمع‌آوری داده‌ها، پرسش‌نامه اطلاعات دموگرافیک، شرح‌حال مامایی و پرسش‌نامه محقق ساخته (17 سؤالی) خودکارامدی زنان در زایمان طبیعی بود که در سه مرحله قبل از آموزش، یک هفته و یک ماه پس از آموزش توسط مادران تکمیل شد. داده‌ها با استفاده از آمار توصیفی و آزمون Mann-Whitney در نرم‌افزار SPSS مورد تجزیه و تحلیل قرار گرفت. یافته‌ها: بر اساس نتایج آزمون Mann-Whitney، نمره خودکارامدی قبل از مداخله در دو گروه تفاوت معنی‌داری نداشت (51/0 = P)؛ در حالی ‌که یک هفته بعد از انجام مداخله (05/0 = P) و یک ماه پس از انجام مداخله (04/0 = P)، تفاوت معنی‌داری بین دو گروه مشاهده شد. نتیجه‌گیری: کلاس‌های آموزش زایمان فیزیولوژیک، در ارتقای نمره خودکارامدی زنان در زایمان طبیعی و انتخاب روش زایمان مؤثر می‌باشد.

Title: بررسی تکرار، شدت و دیسترس ناشی از نشانه‌ها در بیماران مبتلا به لوسمی بستری در بخش‌های هماتولوژی- انکولوژی بیمارستان‌های منتخب شهر اصفهان در سال 1395

http://hsr.mui.ac.ir/index.php/jhsr/article/view/2919

مقدمه: مدیریت نشانه‌ها یک مسأله مهم مراقبتی بین بیماران مبتلا به لوسمی است. با این حال، توجه اندکی به نشانه‌ها از جنبه‌های مختلف (میزان تکرار، شدت و دیسترس) در این گروه از بیماران شده است. روش‌ها: مطالعه حاضر از نوع توصیفی- تحلیلی بود. 400 نفر از بیماران مبتلا به انواع لوسمی بستری در بیمارستان‌های الزهرا (س)، سیدالشهدا (ع) و حجتیه شهر اصفهان در سال 1395 به روش نمونه‌گیری آسان وارد مطالعه شدند. جهت جمع‌آوری داده‌ها از پرسش‌نامه‌های مشخصات دموگرافیک و مقیاس سنجش نشانه‌های یادآمد (Memorial Symptom Assessment Scale یا MSAS) استفاده گردید. داده‌ها با استفاده از روش‌های آمار توصیفی در نرم‌افزار SPSS مورد تجزیه و تحلیل قرار گرفت. یافته‌ها: نشانه‌ها از لحاظ بیشترین شیوع در بیماران (با بیش از 50 درصد شیوع) به «کمبود انرژی، اختلال خواب، احساس غمگینی، درد، عصبی بودن، کسالت داشتن، دل‌نگرانی، احساس تحریک‌پذیری، کاهش اشتها، بی‌حسی و مورمور شدن دست ‌و پا، کاهش وزن، تعریق و خشکی دهان» اختصاص داشت. از نظر میزان تکرار، شدت و دیسترس نیز شامل «دل‌نگرانی، کمبود انرژی، درد، اختلال خواب، احساس غمگینی، عصبی بودن و احساس تحریک‌پذیری» بود. نتیجه‌گیری: نشانه‌های مربوط به بعد روان‌شناختی بیشتر از نشانه‌های بعد جسمی در بیماران مبتلا به لوسمی تجربه می‌شود. بنابراین، نیاز است حمایت‌های روحی- روانی، مشاوره‌ای و مددکاری بیشتری برای این بیماران در نظر گرفته شود.

Title: بررسی فرهنگ ایمنی بیمار در یکی از بیمارستان‌های شهر شیراز در سال 1394

http://hsr.mui.ac.ir/index.php/jhsr/article/view/2875

مقدمه: توسعه فرهنگ ایمنی، عنصر اصلی بسیاری از برنامه‌های ایمنی بیمار در بیمارستان‌ها و مراکز درمانی به شمار می‌رود. هدف از انجام مطالعه حاضر، بررسی فرهنگ ایمنی بیمار در یکی از بیمارستان‌های شهر شیراز بود. روش‌ها: این مطالعه از نوع توصیفی- مقطعی بود که بر روی 210 نفر از کارکنان بالینی یکی از بیمارستان‌های شهر شیراز انجام شد. ابزار پژوهش، ویرایش فارسی پرسش‌نامه اصلاح شده Stanford (Modified Stanford Instrument یا MSI) بود که پس از تأیید روایی و پایایی (74/0 = α) مورد استفاده قرار گرفت. فرهنگ ایمنی بیمار در چهار سطح ضعیف (125-101)، متوسط (150-126)، خوب (175-151) و عالی (200-176) طبقه‌بندی گردید. داده‌ها در نرم‌افزار SPSS مورد تجزیه و تحلیل قرار گرفت. یافته‌ها: میانگین سن و سابقه کار مشارکت کنندگان به ترتیب 68/11 ± 60/28 و 68/11 ± 80/4 سال بود. میانگین نمره فرهنگ ایمنی در مطالعه حاضر 806/0 ± 140/150 به دست آمد. از میان متغیرهای مورد بررسی، ارتباط معنی‌داری بین نمره فرهنگ ایمنی با شغل، بخش‌های مختلف بیمارستان و سن مشاهده شد (050/0 > P). بیشترین سطح فرهنگ ایمنی نیز به ترتیب به بخش‌های آزمایشگاه، رادیولوژی و جراحی اختصاص داشت. نتیجه‌گیری: اگرچه وضعیت فرهنگ ایمنی بیمار در بیمارستان مورد بررسی در سطح خوبی ارزیابی گردید، اما به منظور ارتقای آن به سطح عالی، ضروری است مداخلات مورد نیاز از جمله آموزش مداوم کارکنان و بهبود مستمر نظام حاکمیت بالینی توسط بیمارستان برنامه‌ریزی و اجرا گردد.

Title: بررسي ارتباط ميان سرمايه اجتماعي و تعهد سازماني در يك محيط بيمارستاني

http://hsr.mui.ac.ir/index.php/jhsr/article/view/2844

مقدمه: تعهد سازمانی کارکنان بيمارستان به دليل ويژگي‌هاي منحصر به فرد اين سازمان، از عوامل استراتژیک در عملکرد و خدمت‌رسانی آن‌ها به شمار می‌رود. یکی از مفاهیم اجتماعی جدید در بیمارستان‌ها که می‌تواند تقویت کننده تعهد سازمانی باشد، سرمایه اجتماعی کارکنان است. از اين‌رو، تحقيق حاضر با هدف تعيين ارتباط ميان سرمايه اجتماعي و تعهد سازماني كاركنان در يك محيط بيمارستاني در تهران انجام شد. روش‌ها: در اين مطالعه توصيفي- همبستگي، 337 نفر از کارکنان بخش‌هاي تخصصي يكي از بیمارستان‌هاي تهران شامل پزشكان، پرستاران و دستياران شركت كردند كه با روش نمونه‌گیری طبقه‌بندی شده نظام‌دار و تصادفی ساده وارد مطالعه شدند. ابزار جمع‌آوری داده‌ها، پرسش‌نامه تعهد سازمانی Allen و Meyer و پرسش‌نامه محقق ساخته سرمایه اجتماعی بود که اعتبار آن با استفاده از اعتبار صوری و پایایی آن با استفاده از ضریب Cronbach¢s alpha تأیید گرديد. داده‌ها در نرم‌افزار SPSS مورد تجزیه و تحلیل قرار گرفت. 05/0 > P به عنوان سطح معني‌داري داده‌ها در نظر گرفته شد. یافته‌ها: همبستگی مثبت و معنی‌داری بین سرمایه اجتماعی و تعهد سازمانی (001/0 = P) و همچنین، بعد ساختاری سرمایه اجتماعی و تعهد سازمانی (030/0 = P) مشاهده شد. بین بعد شناختی سرمایه اجتماعی و تعهد سازمانی همبستگی ضعیف و معنی‌داری وجود داشت (280/0 = P). همبستگی بین بعد ارتباطی سرمایه اجتماعی و تعهد سازمانی نیز مثبت و معنی‌دار بود (001/0 > P). نتیجه‌گیری: سرمایه اجتماعی در یک سازمان و یا یک بخش از آن، مي‌تواند منجر به تقویت تعهد سازمانی کارکنان آن سازمان يا آن بخش گردد و كاركناني كه داراي سرمايه اجتماعي قوي‌تري هستند، از تعهد سازماني بالاتري برخوردار مي‌باشند.

Title: بررسی رابطه هوش هیجانی با سلامت عمومی، خودکارامدی، خودتنظیمی و عزت نفس دانش‌آموزان دبیرستان‌های دولتی شهر اصفهان

http://hsr.mui.ac.ir/index.php/jhsr/article/view/2945

مقدمه: اهمیت توجه به هوش هیجانی در نوجوانان نسبت به گذشته افزایش چشمگیری داشته است و باید مؤلفه‌های گوناگون آن مانند سلامت عمومی، خودکارامدی، خودتنظیمی و عزت نفس تقویت گردد. هدف از انجام مطالعه حاضر، تعیین ارتباط میان هوش هیجانی با سلامت عمومی، خودکارامدی، خودتنظیمی و عزت نفس دانش‌آموزان پایه دوم متوسطه دبیرستان‌های دولتی شهر اصفهان بود. روش‌ها کار: این مطالعه از نوع توصیفی- تحلیلی بود که به روش مقطعی بر روی 240 دانش‌آموز دوره دوم متوسطه دوم شهر اصفهان در سال 1394 انجام گردید. آزمودنی‌ها به روش نمونه‌گیری تصادفی چند مرحله‌ای انتخاب شدند و پرسش‌نامه‌های دموگرافیک، هوش هیجانی Shiring، سلامت عمومی Goldberg، خودتنظیمی، خودکارامدی عمومی Sherer (Sherer General Self-Efficacy Scale یا SGSES) و عزت نفس Coopersmith (Coopersmith Self-Esteem Inventories یا CSEI) را تکمیل نمودند. داده‌های جمع‌آوری شده با استفاده از آزمون‌های ضریب همبستگی Pearson، Spearman و Independent t در نرم‌افزار SPSS مورد تجزیه و تحلیل قرار گرفت. یافته‌ها: بر اساس آزمون همبستگی، هوش هیجانی با خودکارامدی (001/0 = P)، عزت نفس (001/0 = P) و سلامت عمومی (001/0 = P) ارتباط معنی‌داری داشت، اما ارتباط آن با خودتنظیمی معنی‌دار نبود (221/0 = P). نتیجه‌گیری: توجه به بهبود هوش هیجانی با توجه به جنسیت می‌تواند در بهبود مهارت‌های خودکارامدی، عزت نفس، سلامت عمومی و مهارت خودتنظیمی برای تدوین برنامه‌های آموزشی دانش‌آموزان حایز اهمیت باشد.

Title: شبیه‌سازی سینتیک ستون‌های کربن فعال گرانولی (GAC) در جذب کروم شش ظرفیتی از جریان پیوسته آب

http://hsr.mui.ac.ir/index.php/jhsr/article/view/2812

مقدمه: با توجه به اثرات سرطان‌زایی و جهش‌زایی و عوارض کلیوی، کبدی و ریوی کروم شش ظرفیتی، بررسی روش‌های حذف آن از آب آشامیدنی ضروری می‌باشد. در این میان، هرچند فرایند جذب سطحی به عنوان یکی از روش‌های مؤثر در حذف کروم معرفی شده، اما تاکنون مطالعه‌ای داخلی در مورد شبیه‌سازی فرایند جذب سطحی با کربن فعال گرانولی (Granular activated carbon یا GAC) در حذف کروم شش ظرفیتی با استفاده از مدل Bohart-Adams صورت نگرفته است. از این‌رو، هدف از انجام پژوهش حاضر، شبیه‌سازی سینتیک ستون‌های GAC در جذب کروم شش ظرفیتی از جریان پیوسته آب بود. روش‌ها: در این مطالعه تجربی- مداخله‌ای، برای تعیین ضریب سرعت و ظرفیت جذب ستون‌های GAC در حذف کروم شش ظرفیتی از جریان پیوسته آب، از مدل Bohart-Adams استفاده شد. در این راستا، سه ستون GAC در مقیاس آزمایشگاهی با عمق بستر 31، 45 و 62 سانتی‌متر در بارگذاری‌های 41/1، 43/2 و 85/3 میلی‌لیتر بر سانتی‌متر مربع در دقیقه برای تصفیه جریان پیوسته آب حاوی 20 میلی‌گرم بر لیتر کروم شش ظرفیتی، مورد بهره‌برداری قرار گرفت. زمان کارکرد ستون‌ها تا لحظه تأمین حداکثر غلظت قابل قبول کروم در آب خروجی در نظر گرفته شد. برای تعیین غلظت کروم از روش استاندارد دی‌فنیل کاربازاید استفاده گردید. یافته‌ها: با تغییر عمق ستون‌های GAC از 31 به 62 سانتی‌متر، مدت سرویس‌دهی آن‌ها به ازای بار سطحی 41/1 میلی‌لیتر بر سانتی‌متر مربع در دقیقه جهت تصفیه جریان پیوسته آب حاوی 20 میلی‌گرم بر لیتر کروم شش ظرفیتی، از 5/2 به 6 ساعت افزایش یافت. با افزایش بار سطحی به 85/3 میلی‌لیتر بر سانتی‌متر مربع در دقیقه، مدت سرویس‌دهی ستون‌های مذکور به ترتیب 5/0 و 5/1 ساعت کاهش یافت. با تغییر بار سطحی از 41/1 به 85/3، ظرفیت جذب هر لیتر از ستون‌ها، از 31/191 به 57/148 میلی‌گرم بر لیتر کروم کاهش پیدا کرد. این روند با تغییر ضریب سرعت جذب GAC از 005/0 به 01/0 میلی‌گرم بر لیتر در دقیقه مطابقت داشت. نتیجه‌گیری: ستون‌های GAC قادر هستند جریان پیوسته آب حاوی 20 میلی‌گرم بر لیتر کروم شش ظرفیتی را در حد تأمین استانداردهای مربوط تصفیه نمایند.

Title: بررسي تغييرات غلظت بنزن هوا در يك مسير پرترافيك شهر اصفهان از طريق روش نمونه‌برداري غير فعال

http://hsr.mui.ac.ir/index.php/jhsr/article/view/2850

مقدمه: روش میکرو استخراج فاز جامد (Solid-phase microextraction یا SPME) به عنوان یک روش ابتکاری نمونه‌برداری هوا مورد توجه قرار گرفته است. مهم‌ترین مزیت این روش نسبت به سایر روش‌های غیر فعال، عدم نیاز به حلال برای استخراج می‌باشد و می‌تواند جهت اندازه‌گیری ترکیباتی مانند بنزن مورد استفاده قرار گیرد. روش‌ها: در مطالعه حاضر کارایی روش SPME برای اندازه‌گیری گاز بنزن در داخل وسایل نقلیه عمومی مورد بررسی قرار گرفت و با نتایج حاصل از روش پمپ و جاذب مقایسه گردید. در مرحله اول، غلظت‌های مشخصی از بنزن در حجم معین استانداردسازی و نمودار کالیبراسیون آن رسم شد. سپس نمونه‌بردارهای SPME در زمان‌های مشخص در داخل و خارج از اتوبوس نصب و به دستگاه کروماتوگرافي گازي (Gas chromatography یا GC) تزریق شد. بدین ترتیب غلظت متوسط بنزن در هوا قابل اندازه‌گیری بود. یافته‌ها: مقادیر اندازه‌گیری شده بنزن با استفاده از روش SPME در داخل و خارج اتوبوس به ترتیب حداکثر تا 26 و 8 میکروگرم بر مترمکعب بود. داده‌های حاصل از نمونه‌برداری با استفاده از روش SPME با داده‌های به دست آمده از روش معمول با استفاده از جاذب و پمپ آناليز مقایسه گردید. بعد از رسم نمودار، 2R به دست آمده حدود 98/0 بود. نتیجه‌گیری: در فصل بهار در بیشتر مواقع آلودگی هوا از نظر بنزن هم در داخل و هم در خارج از اتوبوس‌ها در سطح استانداردی بود، اما این میزان در فصل تابستان مقداری بیشتر از حد استاندارد بود که این افزایش بیشتر در داخل اتوبوس‌ها مشاهده گردید. نتایج مربوط به اندازه‌گیری با استفاده از روش SPME و روش معمول پمپ و جاذب نزدیکی قابل قبولی را نشان داد

Title: کاربرد پرتو مادون قرمز به جای پرتو خورشیدی در معادله شاخص استرس محیطی در محیط خارج از ساختمان

http://hsr.mui.ac.ir/index.php/jhsr/article/view/2371

مقدمه: در بسیاری از محیط‌های کار، به خصوص در فصل تابستان، ارزیابی استرس حرارتی ضروری است. پژوهش حاضر با هدف امکان‌سنجی کاربرد پرتوهای مادون قرمز به جای شدت پرتو خورشیدی در معادله شاخص استرس محیطی انجام شد. روش‌‌ها: اين مطالعه مقطعي در ماه‌های خرداد تا آبان سال 1392 از ساعت 8 تا 18 در شرایط آب و هوایی شهر اصفهان انجام شد. شدت پرتو مادون قرمز در 4 جهت اصلی و بالا و پایین کره مسی ترمومتر گویسان اندازه‌گیری شد. همچنین، دمای تر، دمای خشک و دمای گویسان اندازه‌گیری گردید. سپس، شاخص دمای تر گویسان (WBGT یاWet bulb globe temperature) و شاخص استرس محیطی بر اساس شدت پرتو مادون قرمز (ESIIR یا Environmental stress index) به جای پرتو خورشیدی محاسبه شد. از آزمون‌های آماری همبستگی Pearson و رگرسیون برای تحلیل داده‌ها استفاده گردید. یافته‌ها: بین شاخص ESIIR و شاخص WBGT در هر 6 جهت همبستگی بالایی وجود داشت (01/0 > P). مقدار ضریب همبستگی بین دو شاخص برای هر 6 جهت برابر 963/0 به دست آمد. برآورد شاخص WBGT از متغیر پیش‌بینی کننده شاخص ESIIR از طریق معادله 427/1 + ESIx 959/0 = WBGT امکان‌پذیر بود (94/0 = 2R). نتيجه‌گيري: برای ارزیابی استرس گرمایی در محیط‌های کاری خارج از ساختمان، شاخص ESIIR که بر اساس دمای خشک، رطوبت نسبی و شدت پرتو مادون قرمز محاسبه شد، برآورد کننده خوبی برای شاخص WBGT است

Title: ارزیابی کارایی حذف آنتراسن از محلول‌های آبی با استفاده از زئولیت طبیعی در مقایسه با نوع اصلاح شده‌ آن

http://hsr.mui.ac.ir/index.php/jhsr/article/view/2928

مقدمه: آنتراسن یکی از انواع هیدروکربن‌های آروماتیک می‌باشد که به عنوان ترکیبی سرطان‌زا برای انسان شناخته شده است. ترکیباتی موسوم به زئولیت نیز در حذف آلاینده‌ها و از جمله هیدروکربن‌های آروماتیک مورد استفاده قرار می‌گیرند. بنابراین، هدف از انجام تحقیق حاضر، مقایسه عملکرد زئولیت‌های طبیعی و اصلاح شده با نانوذرات اکسید مس و آهن در حذف آنتراسن از آب بود. روش‌ها: در این پژوهش، فرایند حذف آنتراسن با کاربرد زئولیت‌های طبیعی و اصلاح شده مورد بررسی قرار گرفت و مقدار جذب محلول‌های مورد نظر با استفاده از دستگاه طیف‌سنجی مرئی- فرابنفش (UV/Visible) اسپکتروفتومتر قرائت گردید. همچنین، تأثیر pH، غلظت اولیه آنتراسن، دوز جاذب و زمان تماس در فرایند جذب بررسی شد. یافته‌ها: با توجه به نتایج آزمایش‌ها، بالاترین راندمان برای حذف آنتراسن در pH اسیدی بود. علاوه بر این، بیشترین راندمان حذف با زئولیت خام در غلظت 10 میلی‌گرم بر لیتر آنتراسن و با استفاده از زئولیت اصلاح شده با نانوذرات آهن صفر ظرفیتی و اکسید مس در غلظت 1 میلی‌گرم بر لیتر به دست آمد. از طرف دیگر، برای زئولیت خام بین میزان دوز جاذب و درصد حذف ارتباطی مشاهده نشد؛ در حالی که بیشترین راندمان با کاربرد 20 گرم بر لیتر زئولیت اصلاح شده با نانوذرات اکسید مس و آهن صفر ظرفیتی به عنوان جاذب حاصل گردید. در نهایت، بیشترین راندمان حذف با کاربرد زئولیت‌ها در زمان 120 دقیقه به دست آمد. مکانیسم ایزوترم جذب نیز از ایزوترم جذب Freundlich تبعیت نمود. نتیجه‌گیری: راندمان حذف با استفاده از زئولیت‌ اصلاح شده با نانوذرات اکسید مس و آهن، بیشتر از راندمان حذف با زئولیت خام می‌باشد، اما بر خلاف کارایی بالا جهت سنجش غلظت باقی‌مانده با استفاده از روش اسپکتروفتومتری، برای بهینه نتایج با کاربرد روش Total organic carbon (TOC)، نتایج نشان می‌دهد که در مجموع کارایی حذف آنتراسن با استفاده از جاذب‌های مورد مطالعه کم است.

Title: ارزیابی محصولات پروبیوتیک و غیر پروبیوتیک لبنی در اصفهان از نظر تعداد لاکتوباسیلوس زنده و وجود لاکتوباسیلوس اسیدوفیلوس

http://hsr.mui.ac.ir/index.php/jhsr/article/view/2135

مقدمه: مطالعه حاضر به بررسی کنترل کیفی محصولات پروبیوتیک عرضه شده در بازار ایران از نظر تعداد گونه لاکتوباسیلوس زنده و مقایسه آن با محصولات لبنی غیر پرووبیوتیک پرداخت. همچنین، سویه‌های جدا شده از نظر ادعای تولید کنندگان مبنی بر استفاده از گونه لاکتوباسیلوس اسیدوفیلوس مورد بررسی قرار گرفت. روش‌ها: در این مطالعه، 14 محصول لبنی پروبیوتیک و 8 فرآورده لبنی غیر پروبیوتیک از بازار تهیه گردید. رقت‌های مناسب از هر نمونه در سرم فیزیولوژی به دست آمد و 100 میکرولیتر از رقت‌ها به شکل کشت سطحی بر محیط آگاردار de Man, Rogosa and Sharpe (MRS) حاوی سیستئین هیدروکلراید تلقیح شد. پس از گرم‌خانه‌گذاری و شمارش کلنی‌ها، از هر نمونه کشت، شش کلنی با خصوصیات مورفولوژیک متفاوت جداسازی شد. ایزوله‌ها در محیط کشت MRS حاوی برموکرزول و سیستئین هیدروکلراید حداقل دو بار خالص‌سازی شدند. جهت تفرق سویه‌های پروبیوتیک از غیر پروبیوتیک، از آزمایش رشد در برابر X-Gal استفاده گردید. یافته‌ها: در محصولات پروبیوتیک، جمعیت لاکتوباسیلوس زنده در ماست، کفیر و پنیر به ترتیب 70/5، 32/6 و 27/6 لگاریتم واحد کلنی در گرم بود. در مقابل، میانگین جمعیت لاکتوباسیلوس در مورد کل نمونه‌های غیر پروبیوتیک مورد بررسی شامل ماست، دوغ و پنیر به ترتیب 22/5، 60/3 و 62/3 لگاریتم واحد کلنی در گرم شمارش شد. از 14 محصول پروبیوتیک، 5 نمونه (35 درصد) واجد باکتری‌هایی با قابلیت رشد در حضور صفرا بودند. نتیجه‌گیری: اگرچه میانگین جمعیت لاکتوباسیلوس زنده در فرآورده‌های پروبیوتیک لبنی نسبت به انواع غیر لبنی بیشتر است، اما این شاخص در بخش عمده‌ای از فرآورده‌های لبنی که با عنوان پروبیوتیک در بازار ایران عرضه می‌شود، با حد استاندارد تفاوت زیادی دارد؛ به ویژه در برخی از برندهای تجاری ماست، میانگین شمارش لاکتوباسیلوس‌های دارای قدرت رشد در حضور صفرا، از حداقل تعیین شده در قوانین پایین‌تر می‌باشد.

Title: ارزیابی اجرای نظام مراقبت عفونت‌های بیمارستانی در بیمارستان‌های تحت پوشش دانشگاه علوم پزشکی اصفهان در شهر اصفهان

http://hsr.mui.ac.ir/index.php/jhsr/article/view/2882

مقدمه: در راستای کنترل و مراقبت عفونت‌های بیمارستانی در ایران، نظام مراقبت عفونت‌های بیمارستانی از سال 1384 در بیمارستان‌ها اجرا گردید. از آن‌جا که ارزیابی این نظام می‌تواند به کنترل عفونت‌های بیمارستانی کمک نماید، مطالعه حاضر با هدف تعیین وضعیت اجرای نظام پیشگفت در بیمارستان‌های شهر اصفهان انجام گردید. روش‌ها: پژوهش حاضر به صورت توصیفی در سال 1394 انجام گرفت. 11 بیمارستان آموزشی و غیر آموزشی وابسته به دانشگاه علوم پزشکی اصفهان به صورت سرشماری وارد مطالعه شد. داده‌ها با کمک سیاهه وارسی جمع‌آوری شد که بر اساس دستورالعمل نظام کشوری مراقبت از عفونت‌های بیمارستانی تنظیم و روایی و اعتبار آن آزمون گردید. سیاهه وارسی مشتمل بر 47 گویه با پاسخ بسته سه گزینه‌ای (بلی، تاحدودی، خیر) بود که با مشاهده مستقیم و استفاده از مدارک و مستندات موجود تکمیل و با بهره‌گیری از شاخص‌های آمار توصیفی و کمک نرم‌افزار SPSS تحلیل شد. یافته‌ها: درصد امتیاز مکتسبه نظام مراقبت عفونت‌های بیمارستانی در بعد سازماندهی ۵۰/۹۶ درصد (مطلوب)، در بعد برنامه‌ریزی و خط مشی ۱۲/۸۲ درصد (مطلوب)، تهیه پخش و انتشار گزارش‌ها ۸۰ درصد (مطلوب)، فرایند مداخله ۷۵ درصد (متوسط)، کنترل و پیشگیری از مقاومت آنتی‌بیوتیک ۵۰/58 درصد (متوسط) و طراحی و برنامه‌های آموزشی ۳۰/۹۶ درصد (مطلوب) بود. نتيجه‌گيري: با توجه به متوسط بودن وضعیت نظام مراقبت عفونت‌های بیمارستانی در ابعاد فرایند مداخله، کنترل و پیشگیری از مقاومت آنتی‌بیوتیکی، اتخاذ راهکارهایی در جهت ممانعت از تجویز آنتی‌بیوتیک بدون انجام آنتی‌بیوگرام، محدود کردن مصرف آنتي‌بيوتيك‌هاي با طیف گسترده، طراحی برنامه‌های بومی مداخله‌ای جهت کاهش میزان عفونت‌ها و سنجش میزان تأثیرات اجرای این راهکارها توصیه می‌شود.

Title: شناسایی مؤلفه‌های توانمندسازی فردی و چابکی سازمانی در دانشگاه علوم پزشکی اصفهان و بررسی روابط بین آن‌ها

http://hsr.mui.ac.ir/index.php/jhsr/article/view/3032

مقدمه: هدف از انجام پژوهش حاضر، شناسایی مؤلفه‌های توانمند‌سازی فردی و چابکی سازمانی در دانشگاه علوم پزشکی اصفهان و بررسی روابط بین آن‌ها بود. روش‌ها: پژوهش به روش توصيفي- همبستگی و بر اساس هدف، از نوع توسعه‌ای- كاربردي صورت گرفت. پژوهش حاضر ترکیبی از مطالعات کتابخانه‌های میدانی و نظرسنجی از خبرگان بود. جامعه آماری پژوهش را کارکنان دانشگاه علوم پزشکی اصفهان در سال 1395 به تعداد 25000 نفر تشکیل داده که با استفاده از فرمول نمونه‌گیری کوکران، اعضای نمونه به صورت تصادفی ساده به تعداد 265 انتخاب گردید. از دو پرسش‌نامه محقق ساخته استفاده شده که در مقیاس لیکرت تدوین و پایا گردید (ضریب Cronbach’s alpha توانمندی فردی = 94/0 و چابکی سازمانی = 97/0) و همچنین، نرم‌افزارهای Amos و SPSS استفاده شد. یافته‌ها: علاوه بر مؤلفه‌های موجود در توانمندسازی فردی (احساس معني‌دار بودن، احساس شايستگي، احساس خودمختاري، احساس تأثيرگذاري، احساس اعتماد) و چابکی سازمانی (رهبری و مدیریت، فرهنگ، نوآوری، استراتژی، تغییر و یادگیری، ساختار سازمانی، فن‌آوری اطلاعات، انسجام و یکپارچگی، استفاده از فرصت‌های بالقوه، ارزش‌های مشترک)، مؤلفه‌های حاصل از مطالعات میدانی و نظرسنجی خبرگان (احساس آرمان‌گرایی، احساس مشارکت، احساس خودکنترلی و احساس خلاقیت) و چندپیشگی، قادر به پیش‌بینی هر یک از متغیرها شد. نتیجه‌گیری: توانمندی فردی در تحقیق کنونی تأثیر مثبت و معنی‌داری بر روی چابکی سازمانی در دانشگاه علوم پزشکی اصفهان دارد.

Title: مقایسه متغیرهای شخصیتی کارگران حادثه دیده و حادثه ندیده در صنایع شهر اصفهان

http://hsr.mui.ac.ir/index.php/jhsr/article/view/2468

مقدمه: حوادث، یکی از مهم‌ترین مشکلات کشورهای توسعه یافته و در حال توسعه محسوب می‌شود. عوامل فردی و ویژگی‌های شخصیتی نیز از مهم‌ترین علت وقوع خطاهای انسانی و حوادث می‌باشند. مطالعه حاضر با هدف مقایسه ویژگی‌های شخصیتی افراد حادثه دیده و حادثه ندیده انجام گرفت. روش‌ها: این مطالعه از نوع توصیفی- تحلیلی بود و به صورت مقطعی در سال 1393 و در 19 صنعت از صنایع شهر اصفهان انجام شد. فرم کوتاه پرسش‌نامه پنج شخصیتی NEO (Neuroticism-Extraversion-Openness Five-Factor Inventory یا NEO-FFI) برای 200 نفر (100 نفر حادثه دیده و 100 نفر حادثه ندیده) تکمیل گردید. داده‌ها با استفاده از آزمون‌ Independent t مورد تجزیه و تحلیل قرار گرفت. یافته‌ها: میانگین سنی و سابقه کاری افراد به ترتیب 39/7 ± 19/33 و 32/6 ± 56/9 سال بود. نتایج آزمون Independent t نشان داد که تفاوت معنی‌داری بین ویژگی‌های شخصیتی روان‌نژندی (020/0 = P)، انعطاف‌پذیری (001/0 = P)، برون‌گرایی (035/0 = P) و مسؤولیت‌پذیری (001/0 = P) در دو گروه افراد حادثه‌ دیده و حادثه ‌ندیده وجود داشت، اما تفاوت معنی‌داری در حیطه شخصیتی دلپذیر بودن (100/0 = P) بین دو گروه مشاهده نشد. نتیجه‌گیری: با توجه به نتایج به دست آمده، می‌توان از روی ویژگی‌های شخصیتی افراد، میزان استعداد آن‌ها برای ایجاد حوادث را پیش‌بینی کرد و از به کار گماردن افراد با استعداد بالا در ایجاد حوادث، در پست‌های حساس ممانعت به عمل آورد.

Title: ارزیابی میزان باقی‌مانده سموم آفت‌کش در نمونه‌های خیار مورد استفاده در واحدهای تولید سالاد شهر اصفهان

http://hsr.mui.ac.ir/index.php/jhsr/article/view/3070

مقدمه: آفت‌کش‌ها نوعی از ترکیبات شیمیایی هستند که جهت کنترل یا حذف آفات در سبزیجات به کار می‌روند. هدف از انجام مطالعه حاضر، ارزیابی میزان سموم آفت‌کش در نمونه‌های خیار مورد استفاده در واحدهای تولید سالاد شهر اصفهان بود. روش‌ها: در این پژوهش، 22 نمونه خیار از سه واحد تولید سالاد شهر اصفهان نمونه‌برداری شد و اندازه‌گیری هم‌زمان 16 سم آفت‌کش مجاز در استاندارد ملی ایران شامل «بروموپروپیلات، پرمترین، پریمیکارب، تترادیفون، دلتامترین، دیازینون، دی‌کلرووس، دیمتوات، فاموکسادون، فنازاکوئین، فن‌پروپاترین، کارباریل، کلرپیریفوس، کلروتالونیل، مالاتیون و متالاکسیل» با استفاده از دستگاه کروماتوگرافی گازی- اسپکترومتری جرمی (Gas chromatography-mass spectrometry یا GC-MS) صورت پذیرفت. یافته‌ها: در مطالعه حاضر، بازیافت مطلوب در روش مورد استفاده جهت تشخیص آفت‌کش‌ها معادل 119-86 درصد و انحراف معیار نسبی کمتر از 22/20 بود. حد تشخیص روش (Limit of Detection یا LoD) و حد تشخیص کمی‌سازی (Limit of Quantification یا LoQ) به ترتیب در فاصله مقداری 91/14-54/3 و 92/49-81/11 میکروگرم بر کیلوگرم بود. در 6 نمونه از 22 نمونه خیار مورد بررسی (27 درصد)، آفت‌کش‌های دی‌کلرووس و کلرپیریفوس بالاتر از مرز حداکثر بیشینه مانده (Maximum Residue Levels یا MRLs) تعیین شده در استاندارد ملی ایران مشاهده شد که به ترتیب در فاصله غلظتی 75/188-00/95 و 771-230 میکروگرم بر کیلوگرم شناسایی گردید. مقدار دو آفت‌کش دیمتوات و متالاکسیل در همه نمونه‌ها در مقدار کمتر از MRLs بود و آلودگی به دیگر سموم نیز در نمونه‌ها مشاهده نشد. نتیجه‌گیری: آفت‌کش‌های دی‌کلرووس و کلرپیریفوس در یک سوم از نمونه‌های خیار مورد بررسی با مقدار بیش از MRLs تعیین شده در استاندارد ملی ایران شناسایی گردید. با توجه به اهمیت استفاده از سبزیجات در سبد غذایی روزانه جامعه و همچنین، تأثیرات نامطلوب سموم آفت‌کش بر سلامت جامعه، پایش منظم این گروه از ترکیبات شیمیایی در سبزیجات ضروری به نظر می‌رسد. بنابراین، پیشنهاد می‌شود برنامه‌ریزی لازم در خصوص پایش سموم آفت‌کش در ارگان‌های نظارتی و متولی ‌سلامت صورت پذیرد.

Title: امکان‌سنجی بازیابی آب از پساب حاصل از تأسیسات واحد استریلیزاسیون در بیمارستان آموزشی الزهرا تابعه دانشگاه علوم پزشکی اصفهان

http://hsr.mui.ac.ir/index.php/jhsr/article/view/2820

مقدمه: کمبود آب به خصوص آب شیرین، بازیافت آن را در تأسیسات مختلف الزامی نموده است. از این‌رو، هدف از انجام مطالعه حاضر، امکان‌سنجی بازیابی آب حاصل از پساب تأسیسات واحد استریلیزاسیون بیمارستان آموزشی الزهرا تابعه دانشگاه علوم پزشکی اصفهان جهت استفاده مجدد آب در بویلرهای بیمارستان بود. روش‌ها: در این مطالعه توصیفی- تحلیلی، آزمایش‌های میکروبی شامل HPC (Heterotrophic plate count) و شیمیایی آب شامل سختی کل، هدایت الکتریکی، کل جامدات محلول، pH، کدورت، دما و DO (Dissolved oxygen) بر روی 28 نمونه از آب ورودی و پساب خروجی اتوکلاوهای بیمارستان الزهرا اصفهان مورد سنجش قرار گرفت. یافته‌ها: میانگین نتایج حاصل از سنجش پارامترهای مختلف در آب ورودی و پساب خروجی از تأسیسات استریلیزاسیون بیمارستان الزهرای اصفهان به ترتیب شامل سختی کل (0/85 و 5/16 میلی‌گرم در لیتر)، هدایت الکتریکی (µS/cm 0/270 و 9/253)، کل جامدات محلول (4/146 و 8/137 میلی‌گرم در لیتر)، pH (2/7 و 1/7)، کدورت (NTU یا Nephelometric Turbidity Unit) (61/0 و 08/3)، دما (98/23 و 35/73 درجه سانتیگراد)، HPC (cfu/ml 4/8 و 6/16) و DO (8/7 و 8/3 میلی‌گرم در لیتر) به دست آمد. نتیجه‌گیری: کیفیت پساب حاصل از تأسیسات واحد استریلیزاسیون مرکزی بیمارستان الزهرا اصفهان از نظر استانداردهای کیفی قابل قبول بوده و فقط با اقداماتی مانند اتصال فیزیکی لوله خروجی تخلیه به ابتدای واحد استریلیزاسیون و تنظیم pH می‌تواند دوباره به عنوان آب ورودی واحد استریلیزاسیون دایم مورد استفاده قرار گیرد. در این صورت انتظار می‌رود که کاهش چشمگیری در آب مصرفی این واحد درمانی اتفاق افتد. پیشنهاد می‌گردد، به دلیل وضعیت خشکسالی و کمبود آب استان، از این روش جهت سایر بیمارستان‌ها نیز استفاده گردد.

Title: حذف یون‌های مس از پساب صنعتی توسط جاذب هیدروژل بر پایه اکریلیک اسید/ سبوس گندم/ مونت موریلونیت

http://hsr.mui.ac.ir/index.php/jhsr/article/view/3012

مقدمه: جذب سطحی یکی از کارامدترین روش‌های حذف فلزات سنگین به عنوان یکی از مهم‌ترین آلاینده‌های منابع آبی به شمار می‌رود. هدف از انجام مطالعه حاضر، حذف یون‌های مس از پساب صنعتی توسط جاذب هیدروژل بود. روش‌ها: در این مطالعه، جاذب هیدروژل بر پایه اکریلیک اسید، سبوس گندم و نانو ساختار مونت موریلونیت به روش پلیمریزاسیون درجا ساخته شده و کارایی آن در جذب کاتیون‌های مس از محلول‌های آبی مورد بررسی قرار گرفت. سپس، تأثیر پارامترهای مختلف نظیر غلظت اولیه مس، pH و درصد وزنی مونت موریلونیت ارزیابی گردید. در نهایت، رفتار جذبی این جاذب توسط مدل‌های ایزوترم Langmuir و Freundlich بررسی شد. یافته‌ها: افزایش غلظت اولیه یون‌های مس در محلول، باعث افزایش میزان جذب آن‌ها گردید. بیشترین کارایی جاذب در حذف یون مس با مقدار 3 درصد وزنی نانو ساختار مونت موریلونیت در ساختار هیدروژل به دست آمد. جاذب هیدروژل بیشترین مقدار جذب‌ را در pH حدود 7 نشان داد. تطابق داده‌های جذب ایزوترم این جاذب با مدل Langmuir همبستگی مناسبی با ضریب 99/0 = 2R داشت. نتیجه‌گیری: جاذب هیدروژل سنتز شده بر پایه اکریلیک اسید اصلاح شده با نانو ساختار مونت موریلونیت قابلیت مناسبی در حذف فلز مس از پساب صنعتی دارد.

Title: برآورد سطح امنیت غذایی کشور با شاخص نوین امنیت غذایی جهانی (GFSI)

http://hsr.mui.ac.ir/index.php/jhsr/article/view/2961

مقدمه: در دهه‌های اخیر، امنیت غذایی به یک مشکل جهانی تبدیل شده است. از این‌رو، شناسایی و معرفی شاخص جامعی که بتواند تصویر صحیحی از وضعیت امنیت غذایی کشور ارایه نماید، ضروری به نظر می‌رسد. روش‌ها: در پژوهش حاضر، شاخص امنیت غذایی جهانی (Global Food Safety Initiative یا GFSI) برای نخستین بار جهت مطالعه وضعیت امنیت غذایی کشور ایران طی سال‌های 1360 تا 1392 مورد استفاده قرار گرفت. این شاخص در سال 2012 توسط واحد اطلاعات اکونومیست (Economist Intelligence Unit یا EIU) معرفی شد و یک مدل پویای کمی و کیفی متشکل از مؤلفه‌های منحصر به فرد امنیت غذایی در سه زیرشاخص «دسترسی به مقدار کافی و متنوع از مواد غذایی، توان مالی و کیفیت و سالم بودن» می‌باشد. شاخص GFSI در شناسایی عواملی که بر ناامنی غذایی مؤثر هستند، پا را از گرسنگی فراتر نهاده است و بر مؤلفه‌های کیفی نشان دهنده سیاست‌های دولت‌ها تأکید می‌نماید. جهت محاسبه این شاخص، از داده‌های کتابخانه‌ای سطح کلان سازمان خواروبار کشاورزی، بانک جهانی، بانک مرکزی، قانون برنامه و بودجه و وزارت راه و شهرسازی استفاده گردید. کسب امتیاز 1/75 تا 7/89 درصد بهترین وضعیت، 8/57 تا 0/75 درصد وضعیت خوب، 2/44 تا 7/57 درصد وضعیت متوسط و 0/16 تا 1/44 درصد وضعیت بحرانی را نشان می‌دهد. یافته‌ها: وضعیت امنیت غذایی طی دوره مورد بررسی، از یک روند افزایشی همراه با نوساناتی برخوردار بود و در برخی سال‌ها افزایش و در برخی دیگر کاهش داشت؛ به طوری که کمترین سطح امنیت غذایی در سال 1360 با امتیاز 0/28 درصد (وضعیت بحرانی) و بیشترین آن در سال 1389 با 6/71 درصد گزارش شد. سطح امنیت غذایی در آخرین سال مورد مطالعه (سال 1392) با 0/65 درصد، از وضعیت خوبی برخوردار بود. نتیجه‌گیری: با توجه به اهمیت انکار نشدنی امنیت غذایی، لزوم شناسایی وضعیت کشور با شاخص نوین GFSI ضرورت دارد. بر اساس نتایج، وضعیت امنیت غذایی کشور روندی افزایشی را طی می‌کند، اما از بهترین وضعیت این شاخص فاصله دارد که مستلزم تلاش هرچه بیشتر دولت‌مردان در این حوزه است.

Title: تحلیل سوگیری خودگزارش‌دهی در مصاحبه با زنان کرمانی در خصوص سابقه ابتلا به عفونت‌های آمیزشی

http://hsr.mui.ac.ir/index.php/jhsr/article/view/3136

مقدمه: پرسش در خصوص ابتلا به علایم عفونت‌های آمیزشی با روش‌هایی مثل مصاحبه و پرسش‌نامه خودایفا، یکی از راه‌هایی است که می‌توان به‌ طور غیر مستقیم فراوانی این عفونت‌ها در جمعیت عمومی را برآورد کرد. هدف از انجام مطالعه حاضر، بررسی سوگیری خودگزارش‌دهی ابتلا به عفونت‌های آمیزشی روش مصاحبه‌ای در مقایسه با روش پرسش‌نامه بود. روش‌ها: مطالعه به ‌صورت مقطعی در سال 1393 با روش نمونه‌گیری چندمرحله‌ای در جمعیت عمومی زنان شهر کرمان انجام شد. تعداد 248 زن به طور خودایفا پرسش‌نامه استاندارد شده حاوی 12 سؤال در خصوص سابقه ابتلا به علایم مرتبط با عفونت‌های آمیزشی در زمان مطالعه و در شش ماه گذشته را تکمیل کردند و 248 زن از طریق مصاحبه به این سؤالات پاسخ دادند. از شاخص‌های آمار توصیفی و تحلیل رگرسیون لجستیک و نسبت شانس با لحاظ کردن سطح معنی‌داری 5 درصد استفاده شد. یافته‌ها: شانس خودگزارش‌دهی ابتلا به علایم در زمان مطالعه، در روش مصاحبه 23 درصد کمتر از پرسش‌نامه خودایفا بود (0 = P)، شانس خودگزارش‌دهی ابتلا به علایم در شش ماه گذشته در روش مصاحبه 26 درصد بیشتر از پرسش‌نامه خودایفا گزارش شد (001/0 = P). این روند پس از لحاظ کردن متغیرهای مخدوش ‌کننده نیز وجود داشت. متغیر تحصیلات تأثیر بیشتری بر افزایش شانس خودگزارش‌دهی در پرسش‌نامه خودایفا نسبت به مصاحبه داشت (34/1 = OR). نتیجه‌گیری: مصاحبه همواره موجب سوگیری در خود گزارش‌دهی ابتلا به عفونت‌های آمیزشی نمی‌شود، بلکه بستگی به زمان ابتلا افراد (زمان مطالعه، گذشته) دارد. توصیه می‌شود که در زمان تفسیر نتایج به‌ دست‌ آمده در مطالعات به تأثیرات هر دو روش پرسش‌نامه خودایفا و مصاحبه که ممکن است موجب سوگیری‌هایی در گزارش‌دهی ابتلا به علایم شود، توجه شود.

Title: بررسی کارایی کربن فعال پوسته غلاف گیاه مورینگا اولیفرا در حذف فنل از محلول‌های آبی

http://hsr.mui.ac.ir/index.php/jhsr/article/view/2881

مقدمه: به علت پایداری فنل در محیط ‌زیست و آلودگی آب و منابع طبیعی و اثرات بهداشتی ناشی از آن، حذف این ماده از محیط زیست بسیار حایز اهمیت است. بدین منظور، هدف از انجام مطالعه حاضر، بررسی کارایی کربن فعال ساخته‌ شده از غلاف گیاه مورینگا اولیفرا (Moringa Olifera) به عنوان یک جاذب ارزان ‌قیمت در حذف فنل از محلول‌های آبی بود. روش‌ها: در این پژوهش، از پوست غلاف گیاه مورینگا اولیفرا به عنوان منبع کربن در ساخت کربن فعال استفاده گردید. فرایند ساخت کربن فعال شامل آب‌زدایی و زدایش اجزای زاید از سطح غلاف مورینگا اولیفرا و فعال‌سازی دمایی بود. پس از تهیه جاذب کربن فعال، عوامل مؤثر در فرایند جذب سطحی فنل شامل pH محلول، زمان تماس، دوز کربن فعال و غلظت اولیه فنل مورد بررسی قرار گرفت. همچنین، همخوانی نتایج حاصل از پژوهش با ایزوترم‌های Freundlich و Langmuir بررسی گردید. یافته‌ها: بیشترین کارایی جذب فنل در محدوده pH خنثی به دست آمد. با افزایش و یا کاهش pH محلول، کارایی حذف فنل کاهش یافت. همچنین، با افزایش زمان تماس فنل با جاذب کربن فعال از 5 تا 840 دقیقه، کارایی حذف آن از 23 به 94 درصد افزایش داشت. با افزایش غلظت اولیه فنل، راندمان حذف دارای روندی نزولی بود؛ به طوری که با کاربرد 5/0 گرم بر لیتر کربن فعال و غلظت اولیه فنل 5/7 میلی‌گرم بر لیتر، راندمان حذف فنل 76 درصد به دست آمد و در غلظت‌های 10 و 15 میلی‌گرم بر لیتر، راندمان حذف به ترتیب به 71 و 66 درصد کاهش یافت. نتایج حاصل شده بیشترین قرابت را با ایزوترم Freundlich نشان داد و حداکثر ظرفیت جذب کربن فعال بین 5/6 تا 2/7 میلی‌گرم بر گرم در دامنه غلظت اولیه فنل مورد مطالعه به دست آمد. نتیجه‌گیری: کربن فعال ساخته‌ شده از غلاف گیاه مورینگا اولیفرا کارایی بالایی در حذف فنل از محلول‌های آبی دارد. با توجه به این که گیاه مورینگا اولیفرا نوعی گیاه بومی در مناطق جنوبی کشور است و از غلاف این گیاه استفاده خاصی نمی‌شود، می‌توان از غلاف آن به عنوان یک منبع ارزان‌قیمت جهت ساخت کربن فعال استفاده نمود.

Title: امکان‌سنجی کاربرد میدانی روش میکرواستخراج تک قطره در فضای فوقانی (HS-SDME) در نمونه‌برداری فرمالدئید در هوای محیط کار

http://hsr.mui.ac.ir/index.php/jhsr/article/view/2745

مقدمه: فرمالدئید به علت استفاده گسترده در صنایع و مراکز پزشکی و سمیت آن، یکی از مهم‌‌ترین آلاینده‌های هوا شناخته شده و بر همین اساس، نگرانی‌های زیادی در زمینه حساسیت و دقت روش آنالیز این ترکیب به وجود آمده است. مطالعه حاضر با هدف امکان‌سنجی کاربرد میدانی روش میکرواستخراج تک قطره در فضای فوقانی (Head space-Single drop microextraction یا HS-SDME)، در آماده‌سازی نمونه‌های حاوی فرمالدئید جهت آنالیز با گاز کروماتوگرافی (Gas chromatography یا GC) انجام شد. روش‌ها: در این مطالعه تجربی که به صورت مقطعی انجام گرفت، نمونه‌های هوا طبق روش 3500- National Institute for Occupational Safety and Health (NIOSH) از یک شرکت تولید کننده پشم سنگ جمع‌آوری گردید. نمونه‌های مورد بررسی یک بار با روش استاندارد 3500-NIOSH و بار دیگر با روش HS-SDME و GC مورد آنالیز قرار گرفت و در نهایت، مقادیر غلظت فرمالدئید محاسبه شده در دو روش با هم مقایسه شد. یافته‌ها: بر اساس آزمون Paired t، میانگین غلظت فرمالدئید به روش HS-SDME (27/1 میکروگرم بر میلی‌لیتر) به طور معنی‌داری کمتر از مقادیر روش NIOSH (31/2 میکروگرم بر میلی‌لیتر) به دست آمد (001/0 > P). این مقادیر در روش HS-SDME در نمونه‌های با غلظت متوسط (3-1 میکروگرم بر میلی‌لیتر) و زیاد (5-3 میکروگرم بر میلی‌لیتر) کمتر از مقادیر به دست آمده از روش NIOSH بود. در غلظت کم (1-1/0 میکروگرم بر میلی‌لیتر) نیز میانگین غلظت فرمالدئید در روش HS-SDME کمتر بود، اما اختلاف این مقادیر از لحاظ آماری در این محدوده غلظت معنی‌دار نبود (420/0 = P). نتیجه‌گیری: روش HS-SDME می‌تواند در استخراج نمونه‌های فرمالدئید با غلظت‌های کم کاربرد مؤثری داشته باشد، اما کارایی لازم را در غلظت‌های متوسط و زیاد نخواهد داشت.
